# Covid-19 pandemic in north-west Italy: the potential role of meteorology, air pollution and pollens: Giulia Squillacioti

**DOI:** 10.1093/eurpub/ckac129.562

**Published:** 2022-10-25

**Authors:** G Squillacioti, G Scaioli, C Cassardo, G Arduino, C Siniscalco, R Siliquini, R Bono

**Affiliations:** 1 Public Health and Pediatrics, University of Turin, Turin, Italy; 2 Physics, University of Turin, Turin, Italy; 3 DIR Environment, Energy & Territory, Piedmont Region, Turin, Italy; 4 Life Sciences and Systems Biology, University of Turin, Turin, Italy; 5 A.O.U., City of Health and Science of Turin, Turin, Italy

## Abstract

**Background:**

Italy was the first western country severely affected by the Covid-19 pandemic attesting more than 16 million cases since the outbreak began. Po Valley regions have been most afflicted, with Piedmont ranking sixth at 25,899 cases/100,000 inhabitants. Within this area, air dispersion is hampered making Po Valley a recognised air pollution hotspot. We aimed to explore the potential association between the environment and Covid-19 incidence.

**Methods:**

Daily key air pollutants (NO2, NO, CO, O3, PM10, and PM2.5), meteorological parameters (temperature, %humidity, wind speed and solar radiation), pollens and Covid-19 cases were collected from 01/01 to 31/12/2021 in Turin, Italy. This ecological study preliminarily tested correlations (Spearman) between air pollutants and Covid-19 cases.

**Results:**

The Covid-19 pandemic followed a seasonal trend with the highest number of cases (/100,000 inhabitants) in winter and spring (3.1) followed by autumn (1.3) and summer (0.5) (KW test p < 0.0001). Likewise, all air pollutants showed peaks in winter and autumn and sensibly decreased during spring and summer apart from pollens and O3. O3 follows the photochemical processes reaching its peak in the sunniest periods, while pollens undergo their natural vegetative process. Daily Covid-19 cases were positively correlated with daily-averaged NO2 (0.50, p < 0.0001), NO (0.48, p < 0.0001), CO (0.81, p < 0.0001), PM10 (0.36, p < 0.0001), PM2.5 (0.39, p < 0.0001), pollens (0.15, p = 0.073) and inversely with O3 (-0.44, p < 0.0001). We plan future analyses to test the hypothesized association by enhanced models with lagged air pollution variables, with demographic characteristics and meteorological data as potential confounders.

**Conclusions:**

Results from ecological studies may support researchers’ preliminary understanding of the interplay between environment and Public Health issues, including pandemics. A multidisciplinary approach is mandatory to deepen the complexity of this topic across European regions

**Key messages:**

• The Covid-19 pandemic may be associated with environmental conditions and air pollution but further research is needed.

• Atmospheric particulate matter, including aeroallergens, can favour many airborne-related diseases by acting as immune suppressor and/or carrier, but these hypotheses deserve future research.

